# Learning important clinical skills during junior residency in Japan: A Case of Gram Stain

**DOI:** 10.1002/jgf2.317

**Published:** 2020-05-29

**Authors:** Masaru Kurihara, Yasuharu Tokuda

**Affiliations:** ^1^ Department of Hospital Medicine Urasoe General Hospital Okinawa Japan; ^2^ Muribushi Okinawa Center for Teaching Hospitals Okinawa Japan

Over the past 15 years in Japan, postgraduate mandatory clinical residency training has been introduced and junior residents have been required to learn important clinical skills. As a basic clinical skill, Gram stain for sputum and urine specimens provides clinically meaningful information for pathogen‐directed antibiotic therapy in infectious diseases^1^. Thus, preparing this stain and correctly interpreting microscopic findings are important and junior residents should obtain this skill. Physicians of any specialty could use this skill for their long practice, since many hospital physicians in Japan need to make a correct and timely diagnosis about respiratory or urinary tract infections among their inpatients.

Therefore, we conducted a nationwide survey for junior residents about preparation and performance of Gram stain skill. The survey was conducted in January 2020 at the time of the General Medicine In‐Training Examination (GM‐ITE), which has been implemented every year over the last 10 years around the end of academic year. The ethics committee of the Mito Kyodo General Hospital approved the study (No. 18‐37).

There were a total of 6869 residents participating in the GM‐ITE (539 hospitals). Of these, we analyzed responses from 6164 (response rate, 89.7%) who agreed to the participation of the survey. Of these 6,164 residents (postgraduate year‐2 residents, 50%; women 32%), only 363 (6%) residents reported that they prepared Gram stain for themselves most of the time at diagnosis of respiratory or urinary tract infections (Figure 1). The majority (4152; 74%) seldom prepared Gram stain for them. Of these 4152 residents who did not prepare the stain themselves, 933 (21%) residents did not even ask hospital microbiologists to prepare it for the diagnosis.

**Figure 1:**
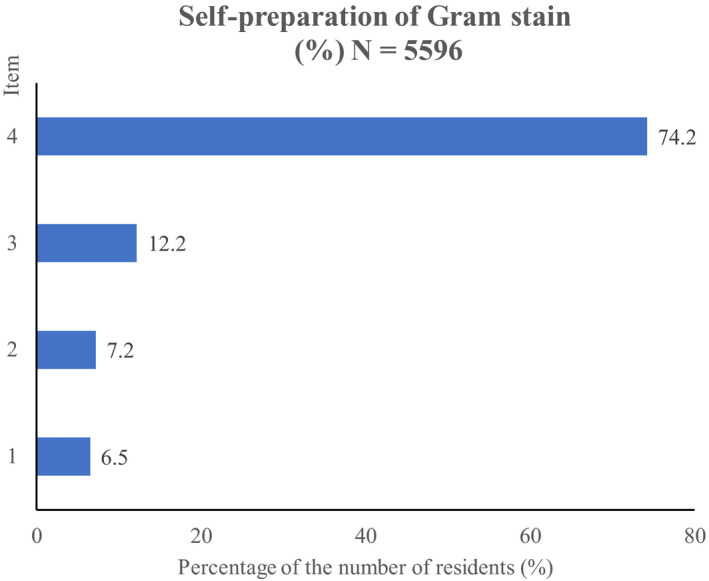
Item 1, use for almost all cases with clinical indication; item 2, use for greater than a half cases; item 3, use for less than a half cases; 4, almost no use

In sputum Gram stain, it is important to identify *Hemophilus influenzae* (H. flu) correctly, since it is most difficult^2^ but it is the most useful finding if accurately interpreted^1^. Thus, in this survey for residents, we asked them about self‐rating for whether residents could confidently interpret sputum Gram stain finding about H. flu. There were only 120 (2.1%) residents who self‐rated they could interpret it with almost complete confidence (Figure 2). The majority (61%) self‐rated they could do it with almost no confidence. Table 1 shows hospital ranking of top 10 based on the higher proportion of the number of residents who could interpret with strong confidence H. flu in sputum Gram stain.

**Figure 2:**
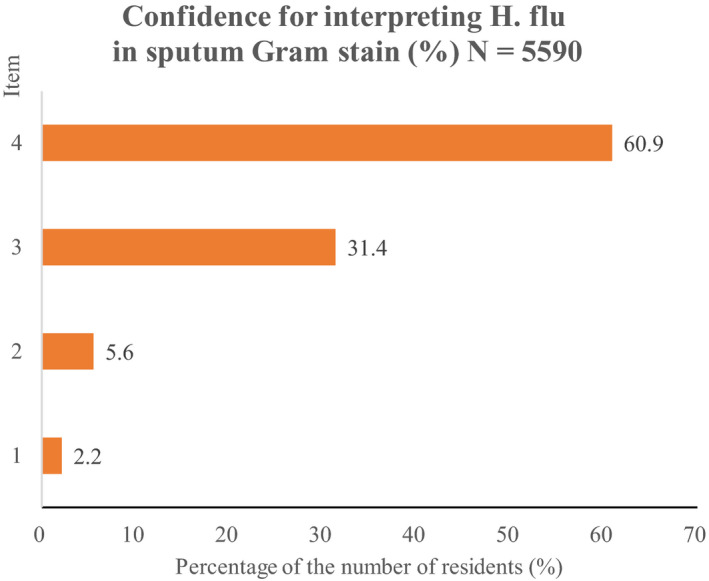
Item 1, strong; item 2, moderate; item 3, weak; 4, no confidence at all. H. flu, *Hemophilus influenzae*

**Table 1:** Top 10 hospitals in Japan by residents with strong confidence for interpreting H. flu in sputum Gram stain

Rank	Hospital	Prefecture	Proportion^a^
1	Okinawa Nanbu Tokushukai Hospital	Okinawa	67
2	Saka General Hospital	Miyagi	28
3	Okinawa Chubu Tokushukai Hospital	Okinawa	27
4	Okinawa Chubu Hospital	Okinawa	22
5	Sakai City General Medical Center	Osaka	20
5	Kawasaki City Ida Hospital	Kanagawa	20
7	Naha City Hospital	Okinawa	12
8	Tsuyama Central Hospital	Okayama	11
8	Sunagawa City Hospital	Hokkaido	11
8	Rakuwakai Otowa Hospital	Kyoto	11

^a^ Percentage of the number of residents with the strong confidence among all residents in each hospital.

It is important to master basic clinical skills for junior residents, and it helps them to perform high‐quality clinical care for patients. Gram stain should not be a lost art of future medicine since it is mandatory to care for patients with infectious diseases. Current epidemics of pneumonia by a novel coronavirus infection (COVID‐19) also makes an accurate diagnosis of pneumonia being greater clinical priority. Teaching hospitals and junior residents in Japan should introduce a learning environment for preparation and interpretation of Gram stain with confidence.

## CONFLICT OF INTEREST

The authors have stated explicitly that there are no conflicts of interest in connection with this article.

## References

[1] Ogawa H , Kitsios GD , Iwata M , Terasawa T . Sputum gram stain for bacterial pathogen diagnosis in community‐acquired pneumonia: a systematic review and bayesian meta‐analysis of diagnostic accuracy and yield. Clin Infect Dis. 2019;9:ciz876. 10.1093/cid/ciz876 PMC7384319 31504334

[2] Fine MJ , Orloff JJ , Rihs JD et al Evaluation of housestaff physicians' preparation and interpretation of sputum Gram stains for community‐acquired pneumonia. J Gen Intern Med. 1991;6(3):189–98. 1712384 10.1007/BF02598958

